# Gigli saw facilitates safe minimal access sagittal suturectomy in infants

**DOI:** 10.3171/2021.1.FOCVID20134

**Published:** 2021-04-01

**Authors:** Robert M. Lober, Shobhan Vachhrajani, Salim Mancho, Kambiz Kamian

**Affiliations:** Divisions of 1 Neurosurgery and; 2 Plastic Surgery, Dayton Children's Hospital, and; Departments of 3 Pediatrics and; 4 Surgery, Wright State University Boonshoft School of Medicine, Dayton, Ohio

**Keywords:** craniosynostosis, endoscopic repair, Gigli saw

## Abstract

The authors describe the use of the Gigli saw for craniectomy in minimal access surgery to address sagittal craniosynostosis. This modification allows for supine positioning and avoidance of potential brain compression with endoscopic instruments, and provides visually clear, safe, and facile removal of the fused suture and surrounding calvaria.

The video can be found here: https://vimeo.com/511568750.

## Transcript

We present “Gigli saw facilitates safe minimal access sagittal suturectomy in infants.” We regularly employ this technique at Dayton Children's Hospital as a collaboration between our Divisions of Neurosurgery and Plastic Surgery.

Craniosynostosis of the sagittal suture results in a narrowed, elongated head shape known as dolichocephaly, characterized by decreased bitemporal width, sagittal ridging, and frontal bossing. Some cases are associated with intracranial hypertension.^1^ Surgery for sagittal synostosis is indicated to normalize head shape and increase cranial volume.^2^

The endoscopic technique introduced by Dr. Jimenez in 1996, which includes a strip craniectomy and barrel stave osteotomies through two small scalp incisions, has gained widespread acceptance because of decreased operative time and blood loss.^2,3^

However, this strategy may be limited by potential difficulties with the Sphinx position and the requirement for specialized endoscopic instruments.

**1:21 Description**. We describe a modification of this technique using a traditional Gigli saw, an instrument that has withstood the test of time in neurosurgery in terms of safety and reliability. We have used this technique at our institution since 2016, and find that it allows for easy positioning in supine position, avoidance of any potential brain compression with endoscopic instruments, and provides visually clear, safe, and facile removal of the fused suture and surrounding calvaria.

**1:50 Surgery Schematic**. This figure demonstrates the basic principle of the technique, in which one surgeon stabilizes the patient's head and protects the infant's skin with a moist sponge, while a second surgeon places the Gigli saw guide and makes the cuts.

**2:02 Patient Positioning**. Patients are positioned supine on a vacuum beanbag, with the head rotated 45° to the right, and the left shoulder and torso are bumped 15°.

We pad all pressure points with cotton and secure the patient before the standard sterile prep and drape.

**2:23 Incision**. After subcutaneous infiltration of local anesthetic, we use a No. 15 blade and a needlepoint Colorado tip cautery to make two transverse, approximately 4-cm incisions, between the anterior and posterior fontanelles.

The subgaleal space is dissected with monopolar electrocautery, with the aid of blunt dissection using the lighted Aufricht retractor.

Care is taken not to violate the fontanelles if they are open.

**2:52 Fontanelle Dissection**. Bipolar electrocautery is applied to periosteum at the anterior edges of the parietal bones. The periosteum curls away nicely from the bone, revealing the interface with the soft fontanelle. A No. 1 Penfield dissector is then used to create a plane under the bone to allow entry of the Gigli saw guide, and then Kerrison rongeurs are used to extend the opening, as needed.

This is usually sufficient for anterior access for the saw, and we generally do not need burr holes anteriorly.

**3:22 Posterior Burr Holes**. Posteriorly, we create burr holes about 1½ inches from the midline on each side. We wait to remove the bone between the burr holes until after the Gigli saw cuts in order to avoid inadvertent detachment, which could lead to bleeding.

**3:39 Suturectomy**. For the suturectomy, one surgeon passes a moist gauze between the incisions and then uses this to retract and protect the scalp, as well as stabilize the head.

The second surgeon gently passes the Gigli saw guide beneath the parietal bone. It passes more easily when initially sweeping in a slightly lateral direction, which also prevents injury to potential bridging veins near the midline.

After the guide is passed, the saw wire is connected and pulled beneath the bone.

The saw wire handles are applied and the cuts are made with just a few strokes of the saw. The moist gauze protects the skin from the saw as it passes through the bone.

The procedure is repeated on the contralateral side. We then use Kerrison rongeurs to remove the bone between the burr holes over the superior sagittal sinus posteriorly. This avoids premature detachment and unexpected bleeding.

**4:38 Bone Removal**. Under direct visualization, the bone can be gently lifted and bridging veins cauterized, if needed.

We also apply gel hemostatic matrix and Gelfoam to the dura before proceeding with additional bony work.

The craniectomy can be extended posteriorly with rongeurs, if needed, to ensure that bone is removed all the way to the lambda.

**5:02 Endscope Inspection**. To demonstrate the craniectomy for this video, we have added an additional step of inspection with the endoscope. Generally, we have adequate direct visualization through the incision using the lighted Aufricht retractor alone.

**5:17 Barrel Stave Osteotomies**. Barrel stave osteotomies are made laterally in the conventional way under direct visualization using Jimenez scissors.

The wounds are then irrigated and the subgaleal space is inspected for hemostasis.

**5:35 Closure**. Finally, we perform a standard closure with interrupted absorbable sutures in the galea and running absorbable sutures in the skin. Using this technique, we have treated 19 patients with a median gestation-corrected age of 3 months, with median follow-up of 15 months. One patient required surgical debridement for a wound infection.

**5:55 Conclusion**. We have found this technique to be safe and feasible with the potential to reduce blood loss and decrease operative time. It avoids brain compression from endoscopic instruments and provides visually clear, safe, and facile dissection between the fused suture and sagittal sinus. Leaving detachment of the bone to the very end prevents inadvertent bleeding during the craniectomy.

It may be performed in the supine position, obviating the difficulties of the Sphinx position, and this technique removes the need for endoscopic assistance.

In the postoperative period, we manage patients no differently than those treated with endoscopic surgery. We begin cranial remodeling helmet therapy after a wound check at 2 weeks, and continue with helmeting for approximately 1 year, depending on cephalometrics.

## Acknowledgments

We would like to thank our surgical assistant Trisha Faris for her dedication and technical expertise.

## Author Contributions

Primary surgeon: all authors. Assistant surgeon: all authors. Editing and drafting the video and abstract: all authors. Critically revising the work: all authors. Reviewed submitted version of the work: all authors. Approved the final version of the work on behalf of all authors: Lober. Supervision: Mancho.

## Supplemental Information

### Previous Presentations

This abstract and operative video demonstration are original and were presented in preliminary form as an electronic poster at the Congress of Neurological Surgeons.
